# Diagnostic value of karyotyping, CMA/CNV-seq, and WES in fetuses with thickened nuchal translucency: perinatal and two-year follow-up outcomes

**DOI:** 10.1186/s12920-026-02331-8

**Published:** 2026-02-21

**Authors:** Mohan Wang, Yizhen Ji, Yasong Xu, Shiyu Sun, Xiaomei Yang, Li Sun, Qichang Wu

**Affiliations:** Prenatal Diagnosis Center, School of Medicine, Women and Children’s Hospital, Xiamen University, No. 10, Zhenhai Road, Xiamen, Fujian 361000 China

**Keywords:** Nuchal translucency, Chromosome karyotype, CMA/CNV-seq, Perinatal outcomes

## Abstract

**Background:**

This study aimed to analyze the perinatal and pediatric outcomes of fetuses with thickened nuchal translucency (NT ≥ 2.5 mm) to enhance prenatal diagnostic strategies.

**Study Design:**

A total of 720 pregnant women with NT ≥ 2.5 mm in the first trimester underwent interventional prenatal diagnosis. These participants were followed up during the perinatal and pediatric periods (2 years after birth).

**Results:**

The detection rate of fetal chromosomal karyotype abnormalities was 32.86%, with trisomy 21 being the most common abnormality. Chromosomal microarray analysis (CMA) and copy number variation sequencing (CNV-seq) increased the detection rate by 10.72%. The most prevalent pathogenic copy number variations (pCNVs) and likely pathogenic copy number variations (lpCNVs) were associated with 22q11.21 microdeletion/duplication syndrome and 15q11.2 microdeletion syndrome, respectively. Excluding pathogenic karyotype abnormalities and pCNV/lpCNVs, the rate of pathological deformities was 19.6%. Whole-exome sequencing (WES) was performed in 11 cases, yielding a detection rate of 72.7% (8/11). There were 339 pregnancy terminations and 381 live births, of which 337 had normal karyotypes, CNVs, and ultrasound results, resulting in a live-birth detection rate of 96.8%. In addition, two cases of psychomotor retardation were identified.

**Conclusion:**

Traditional karyotyping, CMA/CNV-seq testing, and detailed ultrasound examinations are vital diagnostic tools that support genetic counseling for fetuses with thickened NT in the first trimester. Therefore, novel and efficient WES testing is required.

## Key points

• Thickened NT linked to high chromosomal risk.

• CMA/CNV-seq improved detection beyond karyotype.

• WES showed high yield in selected cases.

## Background

Nuchal translucency (NT) refers to fluid accumulation behind the neck of a developing fetus, which can be measured via ultrasound during early pregnancy. It serves as an important indicator of potential chromosomal abnormalities, deformities, fetal anemia, and hypoproteinemia [1–3]. On ultrasound images, the NT appears as an echoless area behind the neck between the deep soft tissue hyperechoic band and skin hyperechoic zone [2]. The fundamental pathophysiological mechanisms underlying NT thickening are complex and not completely understood. The primary cause of NT thickening in fetuses is associated with cardiac anatomical abnormalities. Moreover, abnormalities in the lymphatic system and reflux disorders contribute to heart failure due to congenital malformations and genetic factors [3]. In 1992, Nicolaides et al. first proposed the use of fetal NT thickening in the first trimester as a marker of Down syndrome [4]. However, the International Society of Obstetrics and Gynecology Ultrasound currently lacks a strict definition for the range of NT thickening, and the specific cut-off value for abnormal NT ranges remains controversial in various prenatal diagnostic centers, both domestically and internationally [5]. The British Fetal Medicine Foundation (FMF) defines NT ≥ 3.0 mm as abnormal and recommends invasive prenatal diagnosis, whereas the Society for Maternal-Fetal Medicine (SMFM) in the United States uses NT ≥ 3.5 mm as the threshold for recommending diagnostic testing. Most clinical practices in China currently adopt NT ≥ 2.5 mm as the cutoff.

Recent advances in research and the introduction of novel genetic testing technologies have revealed that NT thickening, even in the absence of chromosomal abnormalities, is associated with adverse pregnancy outcomes. These outcomes include fetal chromosomal microdeletion and microduplication syndromes (MMS), structural anomalies, certain genetic syndromes, intrauterine fetal death, and postnatal neuromotor developmental delays [6]. However, NT thickening is not directly correlated with fetal abnormalities. Research indicates that when fetal karyotype and microdeletion/microduplication tests yield normal results, the complete absence of deformities and growth retardation of the fetus in a systematic ultrasound examination suggests a high probability of a positive pregnancy outcome (> 95%) [7]. NT thickening and subsequent diagnostic examination are major concerns in pregnant women. The intricate association between NT and chromosomal abnormalities necessitates extensive prenatal genetic counseling and individualized diagnostic programs for pregnant women. Currently, informing pregnant women and their families about accurate fetal and pregnancy outcomes is crucial for improving their reassurance, whereas providing clinicians with reliable data to mitigate the residual risk of postpartum issues remains a major challenge in maternal-fetal medicine. However, there is no consensus on the approaches to prenatal diagnosis and management of these conditions. This study retrospectively analyzed 720 singleton cases of interventional prenatal diagnoses associated with an NT of ≥ 2.5 mm in the first trimester. The analysis included copy number variations (CNV), whole-exome sequencing (WES), and fetal karyotype assessments. Additionally, this study examined fetal ultrasound screenings for deformities, tracked pregnancy outcomes, and monitored the growth and development of live infants for up to two years after birth. This study aimed to provide guidance for clinical consultation, prenatal diagnosis strategies, prognostic evaluation, and reproductive planning in cases of increased fetal NT thickness.

## Materials and methods

### Subjects

This study retrospectively analyzed 720 singleton cases of interventional prenatal diagnosis. The cohort comprised 342 primiparous and 378 multiparous mothers who underwent prenatal examinations and ultrasonography between 11 and 13^+ 6^ weeks of pregnancy, revealing a fetal NT ≥ 2.5 mm. Data were collected from January 1, 2018, to December 31, 2021. The average age of pregnant women meeting the inclusion criteria was 31.08 (18–46 years). Pregnancies associated with identified chromosomal abnormalities or significant defects diagnosed prenatally or postnatally were excluded from the analysis. In total, 213 patients underwent transabdominal chorionic villus sampling, 483 underwent transabdominal amniocentesis, and 24 underwent transabdominal umbilical cord blood sampling. Grouped participants based on NT cut-off values of 2.5 mm, 3.0 mm, and 3.5 mm: 220 cases in the 2.5 ≤ NT < 3.0 mm group, 158 cases in the 3.0 ≤ NT < 3.5 mm group, and 242 cases in the NT ≥ 3.5 mm group. All interventional prenatal diagnoses were performed, and the samples were analyzed using G-banded chromosome karyotyping and CMA/CNV-seq detection technology. Among these cases, 11 with normal fetal karyotypes and CMA/CNV-seq results were further tested using WES due to deformities. For fetuses with identified karyotypes or copy number abnormalities, couples are recommended to provide peripheral blood samples to further classify the variants as de novo or genetic. All pregnant women and their families were informed of the indications and risks associated with interventional punctures before surgery and signed an “Informed Consent Form for Surgery.” Following the acquisition of informed consent, the study was approved by the Ethics Committee of Women and Children’s Hospital of Xiamen University.

### Fetal NT measurement standards

Ultrasound measurement of NT thickness is based on the British Fetal Foundation Medical Society (FMF) standards for gestational ages between 11 and 13^+ 6^ weeks or when the fetal crown-rump length is approximately 45–84 mm [8]. In this study, an NT measurement of ≥ 2.5 mm was considered abnormal. High-resolution ultrasound was used, positioning the probe perpendicular to the skin of the fetal neck and back to ensure that the fetus was in a natural propensity position. A median sagittal section was obtained, and the long ribbon-like anechoic area beneath the skin of the fetal neck and back was measured using a Vernier caliper. The measurements were repeated thrice, and the maximum value was recorded as the NT thickness.

### CMA/CNV-seq detection

In this study, CMA was conducted in collaboration with the Beijing Beikang Medical Laboratory, and CNV-seq was performed at the BGI Genomics Medical Laboratory. All participants signed an informed consent form after a comprehensive explanation of the CMA/CNV-seq detection technology. Samples included 5–10 mg of chorionic villi, 10 ml of amniotic fluid, and 2 ml of umbilical cord blood, which were sent for testing.

### Whole exome sequencing

Briefly, 5–10 mg of chorionic villi, 10 ml of amniotic fluid, and 2 ml of umbilical cord blood were collected for testing. Genomic DNA was extracted using a commercial kit. Genomic DNA was fragmented, purified, and labeled to establish the library. A MGISEQ-2000 high-throughput sequencer was used for sequencing with a PE100 + 10 sequencing type, and the original sequencing data were obtained upon completion. Sequences with HG19/HG20 reference genomes were aligned using the Burrows-Wheeler Aligner (BWA) software. The sequence capture effect was concurrently evaluated, and single nucleotide variant (SNV) and insertions/deletions (Indels) were identified using the GATK software to generate the base polymorphism results for the target region. Suspicious mutations were annotated and screened, and Sanger sequencing was performed to verify all identified pathogenic mutations, confirming the microarray capture and high-throughput sequencing results. According to the latest version of the American College of Medical Genetics and Genomics (ACMGG) genetic variation classification standards and guidelines (2015), CNVs were categorized into five groups [9]: benign CNVs (bCNVs), likely benign CNVs (lbCNVs), variants of uncertain significance CNVs (VOUS CNVs), likely pathogenic CNVs (lpCNVs), and pathogenic CNVs (pCNVs).

### The 0-6-year-old children’s neuropsychological development assessment scale

The 0-6-year-old Children’s neuropsychological development assessment scale is a standardized, locally adapted developmental assessment tool in China. It systematically evaluates a child’s developmental level across five core domains: Adaptive Ability (primarily reflecting comprehensive response to external stimuli and problem-solving skills, regarded as an early indicator of intelligence), Gross Motor (assessing postural control and whole-body large muscle activities), Fine Motor (assessing hand-eye coordination and finger manipulation), Language (assessing language comprehension and expression), and Social Behavior (assessing self-care, social interaction, and emotional responses). The assessment results are expressed as a Developmental Quotient, calculated as (Developmental Age / Chronological Age) × 100. In clinical practice, a DQ < 75 in any domain is commonly defined as developmental delay. This scale is used to create a developmental profile of the child, thereby identifying areas of strength and weakness.

### Statistical analysis

Statistical analyses were performed using R software (version 4.2.1). Categorical variables were expressed as counts and percentages. Differences in the detection rates of chromosomal abnormalities and adverse pregnancy outcomes among NT groups (2.5–3.0 mm, 3.0–3.5 mm, and ≥ 3.5 mm) were assessed using Pearson’s chi-square test, followed by post-hoc pairwise comparisons with Bonferroni correction when overall significance was observed. Odds ratios (ORs) and 95% confidence intervals (CIs) were calculated using 2 × 2 contingency tables, with the NT 2.5–3.0 mm group as the reference. All tests were two-sided, and a p value < 0.05 was considered statistically significant, with a Bonferroni-adjusted threshold of *p* < 0.0167 for pairwise comparisons.

## Results

### Chromosome karyotype test results

In this study, 720 samples were subjected to G-banded chromosome karyotyping. Successful cultures were obtained from 715 cases, with 480 demonstrating normal karyotypes and 235 showing abnormal karyotypes, resulting in an abnormality rate of 32.86%. This included 211 cases with abnormal chromosome numbers: 161 cases with abnormal autosome numbers, with trisomy 21 being the most common, followed by trisomy 18 and trisomy 13. There were 50 cases of sex chromosome number abnormalities, with 45 X being the most prevalent. Additionally, 24 cases of structural chromosomal abnormalities were identified; 21 cases had unbalanced structural abnormalities, and three cases had balanced structural abnormalities (Table 1).

**Table 1:** Distribution of karyotypes of 235 cases of NT thickened chromosomal abnormalities

Karyotype type	Number of cases
Abnormal chromosome number	211
Abnormal number of autosomes	161
47, XN, + 21	106
47, XN, + 18	42
47, XN, + 13	12
47, XN, +7	1
Abnormal number of sex chromosomes	50
45, X	40
47, XXX	2
47, XXY	5
47, XYY	3
Chromosome structural abnormalities	24
Balance 46, XN, ?inv (18) (p11.3q11.2) 46, XN, t (1;9) (q42.1;q32) 46, XN, t (7;14) (q31;q13) mat,	3
Imbalance 46, XN, del (1) (q23;q24) 46, XN, del (14) (q24.2q31.2) 46, XN, del (18) (p11.2) dn 46, XN, del (4) (p16.1) 46, XN, del (10) (q26.1) dn 46, XN, del (4) (q13.1q22) 46, XN, del (15) (q11.2q13.1) 46, XN, del (11) (q13.5q23.1) dn 46, XN, del (4) (p15.1) dn 46, XN, del (2) (q36.3) [14]/46, XN [6] 46, XN, ? dup (1) (q32q41) dn 46, XN, der (10) t (10;22) 46, XN, der (8) t (5;8) (p12;p23.1) pat 46, XN, der (1) t (1;4) (p36.2;q31.2) pat 46, XN, der (11) t (6;11) (q23;q25) mat 46, XN, rec (18) dup (18q) inv (18) (p11.32q12.1) mat 46, XN, inv (9) (p11q13), dup (11) (q24q13) 47, XN, +? i (12) (p10) dn 46, XN, i (18) (q10) [34]/46, XN, del (18) (p11.1) [14] 46, X, psu dic (Y) (q11.22 [113]/45, X [10] 47, XN, +mar [33]/46, XN [15] dn	21

47, XN, + 21 contains 4 chimerism, 47, XN, + 18 contains 1 chimerism, 47, XN, + 13 contains 1 chimerism, 45, X contains 6 chimerism, 47, XXX contains 1 chimerism

The detection rates of chromosomal abnormalities and adverse pregnancy outcomes differed significantly among the three NT groups. Chromosomal abnormalities were detected in 24.09% (51/220), 25.32% (40/158), and 53.80% (184/342) of cases in the NT 2.5–3.0 mm, NT 3.0–3.5 mm, and NT ≥ 3.5 mm groups, respectively (χ² = 159.6, df = 2, *p* < 0.001). No significant difference was observed between the two NT < 3.5 mm groups (*p* = 0.78), whereas the NT ≥ 3.5 mm group showed a significantly higher detection rate than both NT < 3.5 mm groups (both *p* < 0.001). Similarly, adverse pregnancy outcomes occurred in 26.82% (59/220), 26.58% (42/158), and 69.59% (238/342) of cases across the three groups, with a significant overall difference (χ² = 207.0, df = 2, *p* < 0.001). Pairwise comparisons showed no difference between the two NT < 3.5 mm groups (*p* = 0.96), but significantly higher rates in the NT ≥ 3.5 mm group compared with both NT < 3.5 mm groups (both *p* < 0.001) (Table 2).

**Table 2:** Association between NT thickness and chromosomal abnormalities and adverse pregnancy outcomes

NT groups	Chromosomal abnormalities *n*/*N* (%)	OR (95% CI)	Adverse pregnancy outcomes *n*/*N* (%)	OR (95% CI)
2.5–3.0 mm	51/220 (24.09)	1.00 (Ref)	59/220 (26.82)	1.00 (Ref)
3.0–3.5 mm	40/158 (25.32)	1.07 (0.66–1.72)	42/158 (26.58)	0.99 (0.63–1.55)
≥ 3.5 mm	184/342 (53.80)	3.86 (2.64–5.63)	238/342 (69.59)	6.24 (4.28–9.10)

### CMA/CNV-seq detection results

CMA/CNV-seq successfully detected abnormalities in 211 cases with chromosomal abnormalities and 21 cases with structural chromosomal imbalances, as identified by karyotype analysis. Additionally, CMA/CNV-seq identified pCNVs in five samples in which karyotype culture failed to identify them. Among 480 cases with normal karyotypes, CMA/CNV-seq detected 23 pCNVs and 24 lpCNVs, resulting in a 10.72% increase in the detection rate compared with karyotyping. 22q11.21 MMS was the most common (26.09%) of the 23 pCNVs, followed by Yq11.223q11.23 microdeletion syndrome (17.39%) (Table 3). Copy number variations associated with neurocognitive disorders were detected in all 24 lpCNV cases, with 15q11.2 microdeletion syndrome being the most common (37.5%), characterized by CNVs ranging from 311.8 kb to 861.33 kb. Other notable CNVs included 16p13.11 MMS (12.5%) with sizes from 1.63 Mb ~ 3.29 Mb, and 22q11.21 MMS (12.5%) ranging from 114.86 kb ~ 1.58 Mb. Two cases of Xp22.31 micro-repeats exhibited CNV sizes of 1.6 Mb (Table 4).

**Table 3:** PCNVs detection in 23 fetuses with thickened NT and normal karyotype

Number	NT mm	Ultrasound results	CNV area and size	CNV involves genes and syndromes/diseases	Outcome
1	4.2	Congenital heart disease	del1p36.33p36.22(10.0 Mb)	Contains 103 OMIM genes including GABRD/1p36 deletion syndrome	Induced abortion
2	2.9	No obvious structural deformity was seen	del3q29q29(1.69 Mb)dn	Contains 21 protein-coding genes including DLG1/3q29 deletion syndrome	Induced abortion
3	3	No obvious structural deformity was seen	del6p25.3p22.2(25.3 Mb) del6q27(2.5 Mb)	Contains 89 OMIM genes including FOXC1/6pter-p24 deletion syndrome Contains 14 OMIM genes including ERMARD/related to AD hereditary periventricular nodular ectopia and other diseases	Induced abortion
4	5	No obvious structural deformity was seen	del7q11.23(1.5 Mb)dn	Contains 26 OMIM genes including ELN/related to Williams-Beuren syndrome (AD)	live birth
5	4.1	No obvious structural deformity was seen	del10p15.3p15.1(4.8 Mb)dn	Contains 9 OMIM genes including ZMYND11/related to autosomal dominant intellectual disability	Induced abortion
6	2.6	Congenital heart disease	dup10p12.2p11.23(5.7 Mb)	Contains 22 OMIM genes including ANKRD26/related to diseases such as thrombocytopenia type 2 (AD)	Induced abortion
7	3.2	No obvious structural deformity was seen	del14q32.12q32.13(1.63 Mb)pat	Contains 22 protein-coding genes/associated with pleuropulmonary blastoma	live birth
8	2.7	Short limbs	Uniparental diploid (UPD) on chromosome 14	Contains DLK1 and other genes/related to Temple syndrome (mat) and Kagami-Ogata syndrome (pat)	Induced abortion
9	3.1	Bilateral polycystic kidney disease	del17q12(1.4 Mb)	Contains 17 OMIM genes including HNF1B/recurrent 17q12 deletion syndrome	Induced abortion
10	2.78	Left polycystic kidney, right hydronephrosis	del17q12(1.5 Mb)dn	Contains 12 OMIM genes including HNF1B/recurrent 17q12 deletion syndrome	Induced abortion
11	2.8	Bilateral polycystic kidney disease	del17q12q12(1.99 Mb)dn	This region contains 26 protein-coding genes/17q12 deletion syndrome	Induced abortion
12	8.09	congenital heart disease	del22q11.21(3.1 Mb)	Contains 44 OMIM genes including TBX1/22q11.2 deletion syndrome	Induced abortion
13	2.9	congenital heart disease	del22q11.21(3.1 Mb)dn	Contains 44 OMIM genes including TBX1/22q11.2 deletion syndrome	Induced abortion
14	4.7	No obvious structural deformity was seen	del22q11.21(2.8 Mb)	Contains 42 OMIM genes including TBX1/22q11.2 deletion syndrome	Live birth with atrial septal defect. At the two-year follow-up, psychomotor and intellectual retardation were noted.
15	2.9	No obvious structural deformity was seen	del22q11.21q11.23(2.6 Mb)	Contains 30 OMIM genes including BCR/22q11.2 distal deletion syndrome	Induced abortion
16	3.37	No obvious structural deformity was seen	dup22q11.21(2.8 Mb)dn	Contains 41 OMIM genes including CLTCL1/22q11.2 microduplication syndrome	Induced abortion
17	3.94	No obvious structural deformity was seen	dup22q11.21q11.21(2.25 Mb)	This region contains 37 protein-coding genes/22q11.2 duplication syndrome	Induced abortion
18	2.8	Intrauterine growth retardation	dupXp22.31(1.6 Mb)	Contains 4 OMIM genes including STS/related to clinical phenotypes such as ichthyosis, global developmental delay, autism, and epilepsy	Live birth. The infant exhibited psychomotor developmental delay at two years of age.
19	7.82	Neck hygroma, generalized edema	delXp22.33q28(155.27 Mb)	Associated with Turner syndrome	Induced abortion
20	5.7	Neck hygroma, generalized edema	delYq11.223q11.23(2.25 Mb)	Contains 17 protein-coding genes including AZFc and DAZ regions/Y-linked spermatogenesis disorder	Induced abortion
21	3.49	No obvious structural deformity was seen	delYq11.223q11.23(3.11 Mb)	This region contains 13 protein-coding genes/Y-linked spermatogenesis disorders.	live birth
22	3	No obvious structural deformity was seen	delYq11.223q11.23(2.16 Mb)	Contains 7 protein-coding genes including BPY2/Y-linked spermatogenesis disorder	live birth
23	3.1	No obvious structural deformity was seen	delYq11.223q11.23(3.55 Mb) pat	Contains 10 protein-coding genes including BPY2/Y-linked spermatogenesis disorder	live birth

**Table 4:** Detection results of LPCNVs in 7 fetuses with thickened NT and normal karyotype

Number	NTmm	CNV area and size	CNV involves genes and syndromes/diseases	Outcome
1	6.4	dup4q22.1(1.8 Mb)	Contains 3 OMIM genes including SNCA/related to diseases such as dementia with Lewy bodies (AD)	Induced abortion
2	3.2	del5p15.33p15.33(1.05 Mb)dn	18 protein-coding genes, including the SDHA gene, are related to diseases such as delayed optic atrophy (AD)	Induced abortion
3	4	dup5p15.1p14.1(8.6 Mb)	Contains 5 OMIM genes including CDH18/related to clinical phenotypes such as autism, global developmental delay, and epilepsy	live birth
4	4.3	dup6q25.3(2.4 Mb)	Contains 10 OMIM genes including ARID1B/related to CoffinSiris syndrome (AD) disease, and clinical phenotypes include intellectual disability, alopecia, sensorineural deafness, etc.	Induced abortion
5	2.9	dup7p21.3(3.9 Mb)	Contains TMEM106B and other 5 OMIM genes/related to hypomyelination leukodystrophy 16 (AD) disease, clinical phenotypes include intellectual disability, learning disabilities, epileptic seizures, gait ataxia, etc.	Induced abortion
6	3.24	del20p12.3(1.4 Mb)	Involving PLCB1 and other 3 OMIM genes/associated with early infantile epileptic encephalopathy type 12 (AR)	live birth, Grade II cleft palate (neonatal)
7	3.3	delXq28(376.0Kb)	Containing part of the AFF2 gene segment/FRAXE type intellectual disability (X-linked recessive), clinical phenotypes include mild to moderate intellectual disability, cognitive difficulties, communication disorders, ADHD, autistic behavior, etc.	live birth

### Excluding the correlation between pathogenic chromosomal karyotypic abnormalities and pCNVs/lpCNVs, NT thickening and pathological deformities

In this study, pathogenic chromosomal karyotype abnormalities and pCNV/lpCNVs were excluded, and 85 cases of NT-thickened fetal ultrasound examinations were found to have pathological deformities (19.6%). Among them, 24 presented with fetal neck cystic hygroma, generalized skin edema, and bilateral pleural effusion. The remaining 61 patients had deformities, including 17 with multiple malformations and 44 with single malformations. Systemic deformities were classified as follows: 25 cases of cardiac malformations, 19 cases of skeletal system deformities, and 18 cases of thoracic and abdominal deformities, including eight cases of omphalocele, seven cases of gastroschisis, one case of diaphragmatic hernia, one case of duodenal atresia, and one case of an abdominal mass. Additionally, eight cases of renal dysplasia and seven cases of abnormal nervous system development were identified. Cardiac malformations were the most frequently observed structural abnormality. Among the 11 fetuses with pathological deformities tested using WES, eight were found to have pathogenic or possibly pathogenic gene mutations, resulting in a detection rate of 72.7% (8/11). Two patients had similar abnormal ultrasound results on prior examinations. WES identified monogenic diseases consistent with autosomal recessive inheritance. Ultrasonography in three cases showed abnormal skeletal development, and pathogenic genes associated with skeletal dysplasia were detected. The remaining three cases exhibited pathogenic genes linked to abnormal phenotypes (Table 5).

**Table 5:** The karyotype and CMA/CNV-seq of 8 fetal cases with thickened NT were normal and WES detected pathogenic/suspected pathogenic variants

Number	NT mm	Ultrasound test results	Adverse reproductive history	Mutated genes/associated diseases/inheritance patterns
1	3.4	Short limb deformity, narrow chest and large abdomen	None	FGFR3:c.2420G > T(p.807Leufs*?) dn Lethal osteoplasia/AD
2	12.7	Generalized skin edema and bone dysplasia	None	COL2A1:c.3022G > C (p.Gly1008Arg)dn osteoarthritis with mild achondroplasia/Spondyloepiphyseal dysplasia, Stanescu type /AD
3	5.4	Neck hygroma, systemic skin edema, short limbs, abnormal intracardiac structure	None	SLC26A2:c.2018 A > T(p.Asp673Val) heterozygous (inherited from father) SLC26A2:c.2046G > A(p.Leu682Leu) heterozygous (inherited from mother) Deformative bone dysplasia, chondrogenesis type 1B/AR
4	4.79	Omphalocele, ventricular septal defect	Similar ultrasound abnormalities recur	PIGN: c.963G > A(p.Gln321Gln) The fetus is homozygous (inherited from both father and mother) Multiple congenital malformations-hypotonia-epilepsy syndrome type 1/AR
5	7.35	Generalized skin edema	Past unexplained neonatal death	LZTR1:c.742G > A(p.Gly248Arg) (inherited from mother) Noonan syndrome/AD
6	6.9	Generalized skin edema	Similar ultrasound abnormalities occur again	CHRNA1:c.1128delG(p.Pro377Leufs*10) heterozygous (inherited from mother) CHRNA1:c.505T > C(p.Trp169Arg) heterozygous (inherited from father) Multiple fatal pterygium syndrome/AR
7	9	Generalized skin edema	None	KMT2D: c.9769 A > T(p.Lys3257*)dnKabuki syndrome type 1/AD
8	6.98	Omphalocele, general skin edema	None	MAP2K2:c.1073_1074dupCG(p.Asp359Argfs*3)dncardiofacial-cutaneous syndrome type 4/AD

### Analysis of perinatal outcomes of 720 cases of fetal NT thickening

**Fig. 1:**
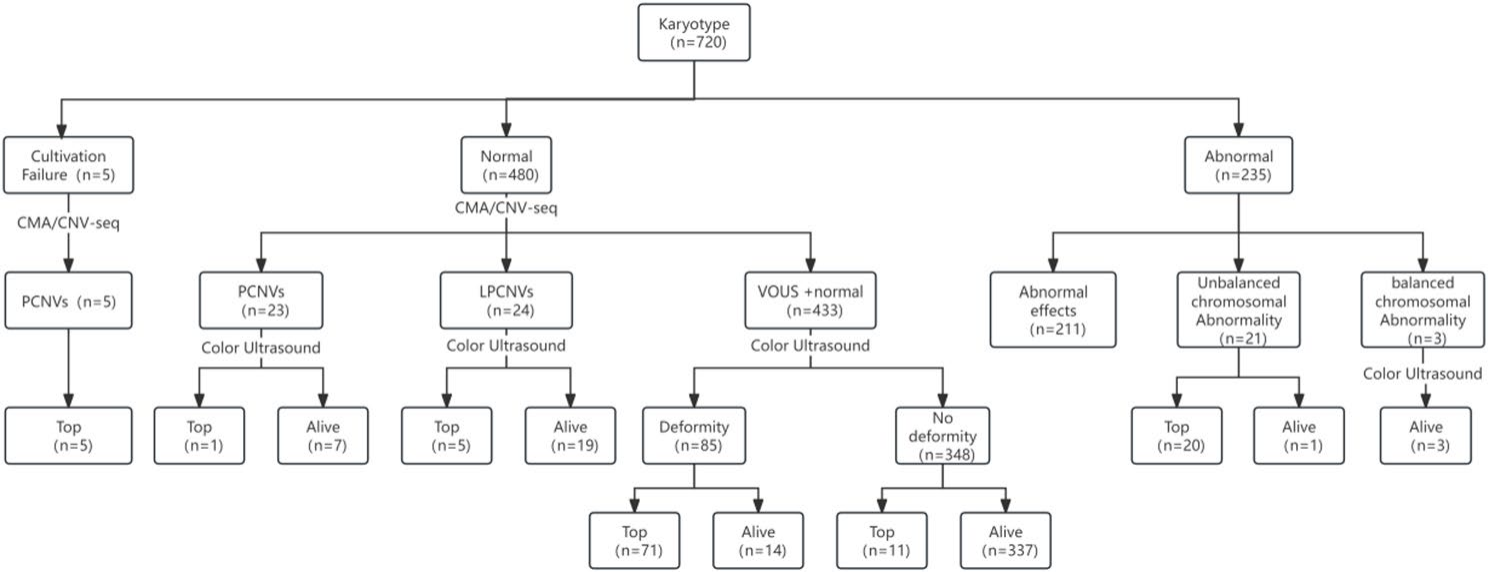
Perinatal outcome analysis of 720 fetuses with thickened NT

Among the 720 singleton pregnancies, there were 339 pregnancy terminations and 381 live births (Fig. 1).

In this study, 211 cases of chromosomal abnormalities and 20 cases of structural chromosomal imbalances diagnosed via karyotype analysis were terminated, accounting for 68.1% of terminations. Additionally, 71 cases without karyotype abnormalities or pCNV/lpCNVs but with pathological deformities detected via ultrasound accounted for 20.9% of the cases. Furthermore, 21 cases of pCNVs (including five cases in which karyotype culture failed) and five cases of lpCNVs detected by CMA/CNV-seq led to pregnancy termination (7.7%). Eleven pregnancies with normal fetal karyotypes, CNVs, and ultrasound results were terminated, accounting for 3.2% of all pregnancies. Among these, five cases were miscarriages, one was a stillbirth, three were diagnosed with severe α-thalassemia, and two cases of premature intrauterine growth restriction and abnormal umbilical blood flow required termination.

Among the 381 live births, 337 had normal fetal karyotypes, CNVs, and ultrasound findings. Of these, 317 were full-term deliveries and 20 were premature births. The birth weight of these newborns was 3367.0 ± 450.6 g, resulting in a live birth rate of 96.8%. The remaining 44 live births included 14 cases of pathological deformities detected via ultrasound, excluding karyotype abnormalities and pCNVs/lpCNVs. There were also 19 cases of lpCNVs, three cases of balanced chromosomal translocations with no ultrasound abnormalities, seven cases of pCNVs, and one case of unbalanced chromosomal translocation.

### Analysis of the follow-up status of 381 live-born infants in early childhood

All live births were followed up in early childhood within the first two years. Trained child health physicians or developmental-behavioral pediatricians conducted the assessments using the standardized Chinese 0-6-year-old Children’s neuropsychological development assessment scale. The actual follow-up compliance rate was 87.40% (333/381). For the 48 children who could not complete the outpatient standardized assessment due to family relocation or personal reasons, we conducted a brief follow-up on their growth and development through structured telephone interviews. Five cases of psychomotor retardation were identified. One case involved del22q11.21 (2.8 Mb), which exhibited no obvious deformities on prenatal ultrasound but was later diagnosed with congenital heart disease (atrial septal defect) after birth. By two years of age, the child exhibited psychomotor retardation and mental impairments. Another case involved dupXp22.31 (1.6 Mb), with prenatal ultrasound showing fetal growth retardation and polyhydramnios. This infant was born full-term via vaginal delivery with a birth weight of 2050 g, and additional WES testing confirmed Noonan syndrome. In another case, prenatal ultrasound suggested a possible partial absence of the corpus callosum, which was later confirmed by ultrasound at six months post-birth, alongside a diagnosis of congenital heart disease (atrial septal defect). WES also suggested Noonan syndrome in this case. Among the 337 live births with normal karyotypes, CNVs, and ultrasound results, one case had an NT measurement of 8.69 mm in the first trimester. WES testing identified a heterozygous mutation (dn) in SETD5 associated with autosomal dominant intellectual developmental disorder-23 (MRD23). Another case, with an NT measurement of 9.1 mm in the first trimester, was found to have a SOS1 gene mutation (dn) associated with Noonan syndrome type 4.

## Discussion

This study involved 720 pregnant women with first-trimester NT measurements ≥ 2.5 mm, each undergoing interventional prenatal diagnosis with follow-up during the perinatal period and early childhood (up to 2 years after birth). The results confirm that early fetal NT thickening is associated with adverse pregnancy outcomes, such as chromosomal abnormalities, chromosomal MMS, structural fetal anomalies, specific genetic syndromes, intrauterine fetal death, and postnatal neuromotor development delays. In this study, the rate of fetal karyotype abnormalities was as high as 32.86%, with trisomy 21 being the most common, highlighting a strong association between fetal NT thickening and chromosomal abnormalities, particularly in Down syndrome. A domestic study using a 2.5 mm NT threshold and amniotic fluid karyotype analysis of 4028 pregnant women reported a chromosomal abnormality rate of 22.02% among fetuses with NT thickening, which was significantly higher than that in fetuses without NT thickening, with trisomy 21 being the most prevalent [10]. Our study also found that when NT thickening was ≥ 3.5 mm, the detection rates of fetal chromosomal karyotype abnormalities and adverse pregnancy outcomes were significantly increased, which is consistent with the conclusions of most domestic and international studies.

Moreover, even in the absence of chromosomal abnormalities, fetuses with NT thickening face heightened risks of a wide range of congenital anomalies, including isolated structural defects, genetic syndromes, and neurodevelopmental delays that often manifest in early childhood [11]. Guidelines from the American College of Maternal-Fetal Medicine indicate that invasive prenatal diagnosis is the first-line management strategy for NT ≥ 3.0 mm.A Meta-analysis revealed that CMA technology could detect an additional 5% of pathogenic variants in cases of fetal NT thickening, with 22q11.2 MMS, 10q26.12q26.3 deletions, and 12q21q22 deletions identified as the most frequent pathogenic CNVs [12]. Our results confirmed that after excluding chromosomal karyotype abnormalities, fetal NT thickening was associated with MMS in the second trimester. Compared with karyotype analysis, CMA/CNV-seq increased the abnormal detection rate by 10.72%, with 22q11.21 MMS being the most frequently observed pCNV type. Our detection rate is significantly higher than the 5% reported in the Meta-analysis, which may be related to our NT thickness inclusion criteria and the recent interpretation standards for CNV.As shown in Table 2, only two cases of congenital heart disease were identified prenatally among six cases of 22q11.21 MMS, and one case presented no abnormality on prenatal ultrasound but was later found to have an atrial septal defect. Follow-up until age two revealed psychomotor and mental retardation. The phenotypic presentation of 22q11.21 MMS is highly variable, ranging from multiple birth defects to mild learning disabilities, with neurodevelopmental disorders and behavioral issues being the most commonly reported phenotypes [13].

Additionally, we identified a case of dupXp22.31 (1.6 Mb) in which prenatal ultrasound showed fetal growth retardation. In addition to low birth weight, infants exhibit psychomotor retardation at the age of two years. Xp22.31 microduplications can lead to a variety of clinical phenotypes, including epilepsy, learning difficulties, psychomotor development retardation, and autism [14]. This study documented 24 cases of lpCNVs linked to neurocognitive disorder phenotypes, with 15q11.2 microdeletion syndrome being the most common, followed by 16p13.11 MMS. The 15q11.2 microdeletion is a newly recognized microdeletion syndrome. Furthermore, reports have shown that the 15q11.2 BP1-BP2 region is susceptible to neurological abnormalities and is associated with a series of neurological diseases, such as language and motor delays, epilepsy, and autism [15].

Additionally, the 16p13.1 region is sensitive to neurocognitive disorders, with evidence suggesting that triple-dose involvement contributes to phenotypes such as developmental delay, intellectual disabilities, learning disabilities, and behavioral abnormalities, with a reported penetrance of approximately 10.6% [16]. All 24 patients with lpCNVs continued with their pregnancies and delivered, as ultrasound examinations showed no deformities. Follow-up revealed no psychomotor retardation in these infants by the age of two. Research data in the field of pediatric genetics show that approximately 12% of children with unexplained developmental delays, intellectual disabilities, abnormal mental development, and other neurocognitive disorders may have clinically significant MMS [17]. Therefore, large-sample, multicenter studies with extended follow-ups are essential to clarify the relationship between neurodevelopmental delays in fetuses with NT thickening and CNVs associated with neurocognitive disorders.

With advancements in WES technology, recent studies have reported that when ultrasound screenings indicate fetal NT thickening and rule out karyotype and microdeletion/microduplication abnormalities, continued pregnancy may result in fetal pathological deformities or developmental retardation linked to fetal monogenic diseases. Additionally, developmental retardation may be related to single-gene fetal disorders [18]. In this study, WES was conducted on 11 fetuses that excluded abnormal karyotypes and pCNV/lpCNVs, all of which presented with pathological deformities. Pathogenic or possibly pathogenic gene mutations were identified in eight cases, including two with abnormal ultrasound findings. WES confirmed single-gene disorders consistent with autosomal recessive inheritance. The remaining six cases revealed pathogenic genes associated with malformation phenotypes, indicating that genetic causes of malformation were linked to fetal NT thickening at the single-gene level before birth. Previous studies have shown that WES identified causative variants in 37.5% of fetuses with ultrasound-detected deformities and 4.8% of fetuses with isolated NT thickening [19].

Early studies have also found that approximately 9% of NT-thickened fetuses with normal chromosomes later developed deformities, with 4% experiencing long-term neurodevelopmental delay [20]. These findings suggest that even when standard karyotype and CMA analyses yield normal results, NT thickening may still be associated with neurodevelopmental disorders, even in the absence of deformities. In this study, 337 live-born infants with normal karyotypes, CNVs, and ultrasound results were followed up until two years of age. Pediatricians conducted formal clinical neurodevelopmental assessments and identified psychomotor retardation in two cases with NT measurements > 5.5 mm in the first trimester, which were confirmed as single-gene disorders via WES. Additionally, follow-ups in early childhood identified three more cases of psychomotor retardation among 44 other live-born infants. One case involved partial agenesis of the corpus callosum, and one was diagnosed with Noonan syndrome via WES. The other two cases were associated with MMS: one with del22q11.21 and the other with del22q11.31, respectively. Furthermore, WES analysis of a case with dupXp22.31 also suggested Noonan syndrome. Recent studies have emphasized that traditional karyotyping and CMA/CNV-seq testing may miss certain genetic abnormalities, particularly in fetuses without deformities. In addition, a meta-analysis suggested that in cases of fetal NT thickening with normal karyotypes and CMA, WES can identify pathogenic or potentially pathogenic genetic variants in approximately 8% of cases with fetal NT thickening, supporting the effectiveness of WES as a supplementary diagnostic method [21].

The tiered genetic testing and counseling management pathway established in this study provides a systematic solution for the clinical management of fetuses with thickened nuchal translucency, effectively integrating precise diagnosis with individualized decision-making. The adopted strategy (karyotype analysis + CNV-seq → WES) not only demonstrates favorable cost-effectiveness by utilizing initial screenings as a “gatekeeper” to optimize resource allocation and avoid the indiscriminate use of whole-exome sequencing (WES), but more importantly, it constructs a complete clinical continuum from etiological clarification to risk management. Its core value lies in the systematic identification and management of genetic “uncertainties,” represented by likely pathogenic copy number variants (lpCNVs). Based on this, we developed an “uncertainty management” counseling framework, which emphasizes stratified interpretation and counseling for lpCNVs in conjunction with fetal ultrasound findings. For fetuses with normal ultrasound structures, most families, after being fully informed, tend to view these findings as risk indicators requiring long-term neurodevelopmental monitoring, whereas when lpCNVs coexist with ultrasound abnormalities, their clinical significance is significantly heightened. By objectively explaining the characteristics of incomplete penetrance and phenotypic heterogeneity associated with such variants, providing specific postnatal monitoring and early intervention pathways, and conducting parental verification to clarify genetic origin and recurrence risk, this framework effectively assists families in translating abstract genetic risks into actionable plans. For copy number variant cases where clinical decision-making is particularly challenging, we organize multidisciplinary consultations to comprehensively assess fetal prognosis, thereby aiding families in making reproductive choices that best align with their values. This integrated approach not only enhances diagnostic efficacy but also genuinely advances the practice of family-centered precision medicine.

Our study has several limitations. First, as the cohort was drawn from a single center and consisted exclusively of Chinese Han individuals, the generalizability of our findings may be limited, and caution should be exercised when applying them to populations with different genetic backgrounds, geographic regions, or clinical practice environments. Second, the clinical interpretation of copy number variations (CNVs) is a rapidly evolving field, and current conclusions may require re-evaluation as knowledge advances. Furthermore, the two-year follow-up period may be insufficient to fully elucidate the long-term neurodevelopmental risks associated with CNVs. Additionally, the inability of some participants to complete standardized outpatient assessments due to family relocation or personal reasons may have impacted the completeness of the follow-up data. Therefore, future large-scale, multicenter studies involving diverse populations are necessary to further validate and extend the reliability and generalizability of our conclusions.

## Conclusion

In summary, there is a significant association between fetal NT thickening and various fetal abnormalities, although this does not provide direct evidence of fetal abnormalities. Traditional karyotyping, CMA/CNV-seq testing, and comprehensive ultrasound examinations are essential diagnostic tools for fetuses with thickened NT during the first trimester. These methods support genetic counseling and assist healthcare providers and patients in making informed decisions. More targeted WES testing should be considered to diagnose potential genetic disorders and enhance diagnostic efficiency and consultation quality, particularly in cases with structural ultrasound abnormalities or NT measurements of ≥ 5.5 mm accompanied by other abnormalities.

## Data Availability

The variation data and related clinical information were uploaded to the National Genomics Data Center (https://ngdc.cncb.ac.cn) with accession number GVM001199.

## References

[1] Bilardo CM, Timmerman E, Pajkrt E, Van Maarle M. Increased nuchal translucency in euploid fetuses—what should we be telling the parents? Prenat Diagn. 2010;30:93–102. 10.1002/pd.2396. 20077440 10.1002/pd.2396

[2] Ville Y. Nuchal translucency in the first trimester of pregnancy: ten years on and still a pain in the neck? Ultrasound Obstet Gynecol. 2001;18:5–8. 10.1046/j.1469-0705.2001.00483.x. 11489217 10.1046/j.1469-0705.2001.00483.x

[3] Bilardo CM, Müller MA, Pajkrt E, Clur SA, Van Zalen MM, Bijlsma EK. Increased nuchal translucency thickness and normal karyotype: time for parental reassurance. Ultrasound Obstet Gyne. 2007;30:11–8. 10.1002/uog.4044. 10.1002/uog.4044 17559183

[4] Nicolaides KH, Snijders RJM, Campbell S, Gosden CM, Berry C. Ultrasonographically detectable markers of fetal chromosomal abnormalities. Lancet. 1992;340:704–7. 10.1016/0140-6736(92)92240-G. 1355807 10.1016/0140-6736(92)92240-g

[5] Atzei A, Gajewska K, Huggon IC, Allan L, Nicolaides KH. Relationship between nuchal translucency thickness and prevalence of major cardiac defects in fetuses with normal karyotype. Ultrasound Obstet Gyne. 2005;26:154–7. 10.1002/uog.1936. 10.1002/uog.1936 15977311

[6] De Domenico R, Faraci M, Hyseni E, Di Prima FAF, Valenti O, Monte S, et al. Increased nuchal traslucency in normal karyotype fetuses. J Prenat Med. 2011;5:23–6. 22439071 PMC3279164

[7] Caughey AB, Kuppermann M, Norton ME, Washington AE. Nuchal translucency and first trimester biochemical markers for down syndrome screening: A cost-effectiveness analysis. Am J Obstet Gynecol. 2002;187:1239–45. 10.1067/mob.2002.127144. 12439512 10.1067/mob.2002.127144

[8] Nicolaides KH, Azar G, Byrne D, Mansur C, Marks K. Fetal nuchal translucency: ultrasound screening for chromosomal defects in first trimester of pregnancy. BMJ. 1992;304:867–9. 10.1136/bmj.304.6831.867. 1392745 10.1136/bmj.304.6831.867 PMC1882788

[9] Richards S, Aziz N, Bale S, Bick D, Das S, Gastier-Foster J, et al. Standards and guidelines for the interpretation of sequence variants: a joint consensus recommendation of the American College of Medical Genetics and Genomics and the Association for Molecular Pathology. Genet Sci. 2015;17:405–24. 10.1038/gim.2015.30. 10.1038/gim.2015.30 PMC4544753 25741868

[10] Study on the relationship between the fetal nuchal translucency and chromosome abnormalities. (in Chinese) Chinese Journal of Birth Health & Heredity. 2017;25:53–55 + 48. 10.13404/j.cnki.cjbhh.2017.03.022.

[11] Atzei A, Gajewska K, Huggon IC, Allan L, Nicolaides KH. Relationship between nuchal translucency thickness and prevalence of major cardiac defects in fetuses with normal karyotype. Ultrasound Obstet Gynecol. 2005;26:154–7. 10.1002/uog.1936. 15977311 10.1002/uog.1936

[12] Grande M, Jansen FAR, Blumenfeld YJ, Fisher A, Odibo AO, Haak MC, et al. Genomic microarray in fetuses with increased nuchal translucency and normal karyotype: a systematic review and meta-analysis. Ultrasound Obstet Gyne. 2015;46:650–8. 10.1002/uog.14880. 10.1002/uog.14880 25900824

[13] Tang KL, Antshel KM, Fremont WP, Kates WR. Behavioral and Psychiatric Phenotypes in 22q11.2 Deletion Syndrome. J Dev Behav Pediatr. 2015;36:639–50. 10.1097/DBP.0000000000000210. 26372046 10.1097/DBP.0000000000000210 PMC4586411

[14] Esplin ED, Li B, Slavotinek A, Novelli A, Battaglia A, Clark R, et al. Nine patients with Xp22.31 microduplication, cognitive deficits, seizures, and talipes anomalies. Am J Med Genet Pt A. 2014;164:2097–103. 10.1002/ajmg.a.36598. 10.1002/ajmg.a.36598 24800990

[15] Picinelli C, Lintas C, Piras IS, Gabriele S, Sacco R, Brogna C, et al. Recurrent 15q11.2 BP1-BP2 microdeletions and microduplications in the etiology of neurodevelopmental disorders. Am J Med Genet Pt B. 2016;171:1088–98. 10.1002/ajmg.b.32480. 10.1002/ajmg.b.32480 27566550

[16] Li J, Hojlo MA, Chennuri S, Gujral N, Paterson HL, Shefchek KA, et al. Underrepresentation of Phenotypic Variability of 16p13.11 Microduplication Syndrome Assessed With an Online Self-Phenotyping Tool (Phenotypr): Cohort Study. J Med Internet Res. 2021;23:e21023. 10.2196/21023. 33724192 10.2196/21023 PMC8074853

[17] Nevado J, Mergener R, Palomares-Bralo M, Souza KR, Vallespín E, Mena R, et al. New microdeletion and microduplication syndromes: a comprehensive review. Genet Mol Biol. 2014;37(1 suppl 1):210–9. 10.1590/S1415-47572014000200007. 24764755 10.1590/s1415-47572014000200007 PMC3983590

[18] Äyräs O, Eronen M, Tikkanen M, Rahkola-Soisalo P, Paavonen J, Stefanovic V. Long‐term outcome in apparently healthy children with increased nuchal translucency in the first trimester screening. Acta Obstet Gynecol Scand. 2016;95:541–6. 10.1111/aogs.12878. 26918672 10.1111/aogs.12878

[19] Cao C, Liu F, Yang Y, Zhang Q, Huang J, Liu X. Prenatal whole-exome sequencing in fetuses with increased nuchal translucency. Molec Gen Gen Med. 2023;11:e2246. 10.1002/mgg3.2246. 10.1002/mgg3.2246 PMC10655512 37766479

[20] Miltoft CB, Ekelund CK, Hansen BM, Lando A, Petersen OB, Skovbo P, et al. Increased nuchal translucency, normal karyotype and infant development. Ultrasound Obstet Gyne. 2012;39:28–33. 10.1002/uog.10060. 10.1002/uog.10060 21837765

[21] Di Girolamo R, Rizzo G, Khalil A, Alameddine S, Lisi G, Liberati M, et al. Whole exome sequencing in fetuses with isolated increased nuchal translucency: a systematic review and meta-analysis. J Maternal-Fetal Neonatal Med. 2023;36:2193285. 10.1080/14767058.2023.2193285. 10.1080/14767058.2023.2193285 37019452

